# The secret life of kinases: functions beyond catalysis

**DOI:** 10.1186/1478-811X-9-23

**Published:** 2011-10-28

**Authors:** Jens Rauch, Natalia Volinsky, David Romano, Walter Kolch

**Affiliations:** 1 Systems Biology Ireland, University College Dublin, Belfield, Dublin 4, Ireland; 2 Conway Institute, University College Dublin, Belfield, Dublin 4, Ireland

**Keywords:** kinase, kinase-independent, non-catalytic, ERBB, EGFR, Raf, ERK, Src, PI3K, AKT, IGF, Cell cycle, PAK, PDK, FAK, ASK

## Abstract

Protein phosphorylation participates in the regulation of all fundamental biological processes, and protein kinases have been intensively studied. However, while the focus was on catalytic activities, accumulating evidence suggests that non-catalytic properties of protein kinases are essential, and in some cases even sufficient for their functions. These non-catalytic functions include the scaffolding of protein complexes, the competition for protein interactions, allosteric effects on other enzymes, subcellular targeting, and DNA binding. This rich repertoire often is used to coordinate phosphorylation events and enhance the specificity of substrate phosphorylation, but also can adopt functions that do not rely on kinase activity. Here, we discuss such kinase independent functions of protein and lipid kinases focussing on kinases that play a role in the regulation of cell proliferation, differentiation, apoptosis, and motility.

## Introduction

Kinases are conserved during evolution. Orthologs with 'kinase domains' (so-called protein kinase-like folds; PKL) are found in all three domains of life, [1]. Interestingly, comparing the 'kinomes' from nematodes, insects, and vertebrates a surprising number of kinases are shared. In eukaryotes, the protein kinase family is one of the largest gene families, counting for about 2% of all genes. The human genome contains 518 putative protein and lipid kinases. Based on sequence similarities they can be divided into 9 groups of conventional kinases, which feature a typical kinase domain sequence, and 8 small groups of unconventional kinases, which lack typical kinase domain sequences but reportedly possess biochemical kinase activity [2,3]. Almost half of the human kinases can be mapped to known disease loci, cancer amplicons, and mutations or their deregulation can be directly correlated to human disease. Therefore, it comes as no surprise that kinases are intensively studied, and kinase inhibitors have now a firm place in the pharmaceutical armoury.

The importance of protein phosphorylation is underlined by a Nobel Prize in Physiology or Medicine awarded to Edmond H. Fischer and Edwin G. Krebs in 1992 *"for their discoveries concerning reversible protein phosphorylation as a biological regulatory mechanism"*. Their key discovery about 55 years ago was that the conversion of the inactive enzyme phosphorylase b to the active phosphorylase a is caused by phosphorylation, and that the conversion factor is a protein kinase, phosphorylase kinase [4,5]. That breakthrough has established a firm role for protein kinases in the regulation of diverse fundamental cellular processes and spawned an immensely fruitful field of kinase research. At the same time this success, however, has blinkered us to solely concentrate on the catalytic activities of kinases neglecting other functions of these proteins, which do not require the phosphotransferase activity.

**Figure 1:**
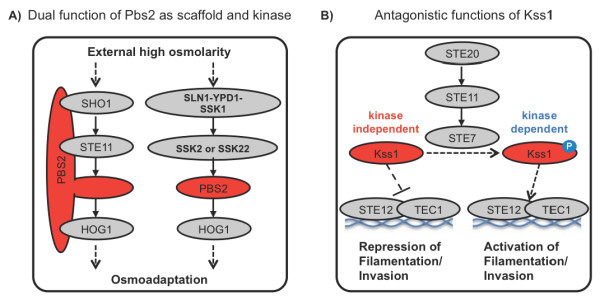
**Examples of catalytic-independent functions in yeast**. **(A) **The dual role of PBS2 as scaffold and kinase in yeast osmo-sensing pathways. **(B) **Antagonistic functions of the kinase Kss1 in filamentation and invasion.

Early examples of non-catalytic functions of protein kinases were discovered in yeast. In 1997 Posas and Saito showed that the yeast Pbs2p protein can serve both as a scaffolding protein and a protein kinase [6]. The adaptation of the yeast *S. cerevisiae *to high osmolarity is regulated by two independent pathways, which both contain a three-tiered cascade of kinases. In one of these pathways Pbs2p serves as a bona fide kinase (MAPKK), which links signalling from SSK2/SSK22 (a MAPKKK) to HOG1 (a MAPK). In the other pathway Pbs2p functions both as a kinase and a scaffold by assembling a complex of Sho1p, Ste11p, and Hog1p proteins and at the same time providing the kinase link between Ste11p and Hog1p (Figure 1A). In another example from budding yeast, Madhani and colleagues showed in 1997 that the MAPK Kss1 has important non-catalytic functions [7]. Non-phosphorylated Kss1 inhibits filamentation and haploid invasion through the kinase independent inhibition of the Ste12-Tec1 transcription factor complex. Phosphorylation by Ste7 (a MAPKK) activates Kss1 catalytic activity and converts Kss1 from a repressor of filamentation into an activator. This second function of Kss1 requires its kinase activity, which acts to stimulate the Ste12-Tec1 complex (Figure 1B).

Although these discoveries are not only early but still some of the clearest examples for a dual function of protein kinases, this new conceptual view did not gain traction until the last few years where a flurry of other examples began to emerge and are now receiving sharply increasing attention.

This review presents a synopsis of protein kinase functions that are independent of catalytic activity, with a special focus on kinases involved in the regulation of proliferation, apoptosis, differentiation, cell adhesion and migration. A comprehensive summary is given in Table 1. Due to space constraints we do not discuss the functions of pseudokinases, but only review recent results suggesting that in some cases their catalytic activities have developed to become highly specialized rather than being lost.

**Table 1:** Catalytic-independent kinase functions according to the 7 major kinase groups.

Kinase Group	Protein	Notes	Organism	References
**Receptor Tyrosine Kinases**	EGFR	Mitogen-activated protein kinase stimulation by a tyrosine kinase-negative epidermal growth factor receptor	CHO (Chinese hamster ovary) cells	[21]

		Tyrosine phosphorylation of mitogen-activated protein kinase in cells with tyrosine kinase-negative epidermal growth factor receptors	B82L cells (mouse)	[19]

		EGFR-ERBB2 oligomers activate ERK and Akt, independent of EGFR kinase activity	human	[24]

		Kinase-negative EGFR retains the capacity to stimulate DNA synthesis	CHO cell line (hamster)	[20]

		No kinase activity of EGFR is required for activation of c-fos expression	mouse	[23]

		Kinase-independent EGFR prevents autophagic cell death by maintaining intracellular glucose level through interaction and stabilization of the sodium/glucose cotransporter 1 (SGLT1).	human	[16]

		EGFR and EGFRvIII interact with PUMA to inhibit mitochondrial translocalization of PUMA and PUMA-mediated apoptosis independent of EGFR kinase activity. This function of EGFR/EGFRvIII leads to tumor drug resistance of glioblastoma.	human	[25]

	ApTrkl	Aplysia Trk-like receptor (ApTrkl), a Trk-like receptor in Aplysia sensory neurons, was shown to have two modes of receptor internalization: kinase activity-dependent internalization and serotonin-dependent, kinase activity-independent internalization	mouse	[213]

	Insulin Receptor	Unliganded insulin- and IGF-1 receptors exert a permissive effect on cell death	Mouse adipocytes	[214]

		Induces phosphorylation and activation of phosphatase PHLPP1, a negative regulator of Akt2 activity		[215]

	Insulin-Like Growth Factor I (IGF-1) Receptor	Mediates Erk1/2 phosphorylation in a tyrosine phosphorylation independent manner.	Smooth muscle cells	[34]

	EphA2	Some of EphA2 functions in cell motility, invasion and bone formation are kinase-independent		[216]

	EphA4/SEK1	Kinase-dependent and kinase-independent functions of EphA4 receptors in major axon tract formation in vivo	mouse	[217]

	EphA8	Overexpression of EphA8 enhances cell attachment to fibronectin		[218]

	EphB/NUK	Kinase independent function of EphB receptors in retinal axon path finding to the optic disc from dorsal but not ventral retina	mouse	[219]

		Kinase-Independent Requirement of EphB2 Receptors in Hippocampal Synaptic Plasticity	mouse	[220]

		EphB2 regulates positioning of differentiated Paneth cells in small intestine independently of kinase activity	intestinal epithelium	[221]

	EphB3	EphB3 is overexpressed in non-small-cell lung cancer and promotes tumor metastasis in a kinase-independent manner.	Lung	[222]

		Overexpression of wild type or kinase dead protein decreases Cdc42/Rac activity and reduces cell migration.		[223]

	EphA3	In the absence of ephrin interaction, kinase-dead EphA3 recruits other Eph molecules for oligomerization	human	[224,225]

	VAB-1	VAB-1, a C. elegans Eph receptor, regulates embryonic development by kinase-dependent and -independent functions	*C. elegans*	[226-228]

	c-kit	Complex formation with granulocyte macrophage colony-stimulating factor (GM-CSF) receptor.		[229]

**Non-receptor Tyrosine kinases**	Src	Overexpresion of non-catalytic domains of Src alters focal adhesion properties		[107]

		c-Src enhances the spreading of src-/- fibroblasts on fibronectin by a kinase-independent mechanism	mouse	[109]

		Activation of the Src-dependent adaptor protein pp130cas in fibroblasts in response to fibronectin binding does not require intrinsic Src kinase activity	mouse	[110]

		Kinase-deficient Src protects src-/- mice against osteopetrosis.	mouse	[112]

		Src mediates B cell antigen Receptor response		[230]

		Src mediates FAK phosphorylation at several tyrosine residues independently of its kinase activity	KM12C (Human colon cancer)	[111]

		Src regulates Jak2/Stat5 activation induced by prolactin in mammary tissue in a kinase-independent manner		[113]

	Brk	Brk (PTK6) promotes breast carcinoma cell proliferation		[231]

	FRK-1	Fer-related kinase-1 (FRK-1) performs a kinase-independent function in differentiation and morphogenesis of the C. elegans epidermis during embryogenesis.	Caenorhabditis elegans	[232]

	Lck	Kinase-independent function of Lck in potentiating antigen-specific T cell activation	human	[114]

	Hck	The Src Family Kinase Hck Interacts with Bcr-Abl by a Kinase-independent Mechanism and Phosphorylates the Grb2-binding Site of Bcr-Abl	COS7 cells	[233]

	Lyn	Negative regulation of B cell Ag receptor (BCR) induced activation of Protein Kinase C (PKC)	Chicken B cells	[115]

		Lyn increases p53 levels and stimulates p53-mediated transcription by a kinase-independent mechanism		[234]

	c-Abl	Promotes p53 DNA binding		[235]

		Negatively regulates UV damaged DNA repair by recruiting CAL-4A ubiquitin ligase		[236]

		c-Abl promotes proteolytic destruction of damaged DNA binding proteins in a kinase-independent manner	mouse	[236]

		Abl proper subcellular localization is correlated with its kinase-independent activity	Drosophila	[237]

	FAK (Protein Tyrosine Kinase 2)	FAK initiates endothelial cell development during embryogenesis	Mouse endothelial cells	[124]

		FAK promotes cell survival by enhancing p53 degradation	Mouse fibroblasts and human cell lines	[126]

		Mediates JNK activation in a kinase-independent manner by recruiting paxillin to the plasma membrane		[118]

	Pyk2 (Protein Tyrosine Kinase 2 B)	Pyk2 facilitates cell growth and survival by limiting p53 levels	MEF and cell lines	[128]

	ACK (TNK2)	The scaffold function of ACK, rather than kinase activity, seems important in the context of cell growth control	human	[238]

	ACK2	Overexpressed ACK2 inhibits kinase activity of FAK and cell growth independently of its kinase activity; however its ability to dissolve actin stress fibers and to disassemble focal complexes requires kinase activity	NIH3T3 cell line	[239]

	BMX	Bone Marrow Kinase (BMX) regulates inflammation in rheumatoid arthritis	mouse	[240]

	Itk	Itk mediates antigen receptor induced activation of transcription factor SRF (Serum Response Factor) independently of its kinase activity	DT40 chicken B cells	[241]

		Itk regulates Vav localization and T cell Receptor-induced actin polarization independently of its kinase activity.	T cells	[242]

	Zap-70	Whereas the kinase activity of Zap-70 is required for signal transduction downstream to the T cell antigen receptor (TCR), this protein has kinase-independent functions in activating small G protein Rap1, required for integrin-mediated adhesion.	T cells	[243]

**TKL**	TβRI	TGFβ receptor recruits and activates TAK1 via interaction with TRAF6	human	[244]

	Raf-1	Raf-1 binds to and inhibits ROK-alpha kinase activity	MEF	[66]

		Raf-1 plays an essential, kinase-independent function as a spatial regulator of Rho downstream signaling during migration.	MEF	[65]

		Raf-1 sets the threshold of Fas sensitivity by modulating ROK-α signaling	MEF	[64]

		Raf-1:ROK-alpha complex linked to STAT3/Myc activation is crucial for cell fate decisions in Ras-induced tumorigenesis.	mouse	[67]

		Raf-1 binds and inhibits the pro-apoptotic kinase MST2	human and mouse cell lines	[68-70,245]

		Raf-1 binds and inhibits the pro-apoptotic kinase ASK1	mouse	[246]

		Cardiac-specific disruption of the Raf-1 gene induces cardiac dysfunction and apoptosis	mouse	[75]

		Raf-1 promotes cell survival by antagonizing apoptosis signal-regulating kinase 1 through a MEK-ERK independent mechanism	human	[74]

		MEK kinase activity of Raf-1 is not essential for function and normal mouse development. Raf-1 plays a role in preventing apoptosis.	mouse	[43,44]

	A-Raf	A-Raf binds and inhibits the pro-apoptotic kinase MST2	human	[71,72]

	Raf-1/B-Raf	Regulation and role of Raf-1/B-Raf heterodimerization	human	[51,53]

		Mixed-lineage kinase 3 (MLK3) regulates B-Raf through maintenance of the B-Raf/Raf-1 complex and inhibition by the NF2 tumor suppressor protein	human	[55]

		Diacylglycerol Kinase η Augments C-Raf Activity and B-Raf/C-Raf Heterodimerization	human	[56]

	PKK	Protein kinase C-associated kinase (PKK, also known as RIP4/DIK) lacking kinase activity can induce partial activation of NFκB		[247]

**CMGC**	ERK	ERK (Extracellular signal-regulated kinase) acts as a transcriptional repressor for interferon gamma-induced genes.	human	[89]

		Catalytic Activation of the Phosphatase MKP-3 by ERK2 Mitogen-Activated Protein Kinase	human	[86]

		ERK Activates Topoisomerase IIalpha through a Mechanism Independent of Phosphorylation	mouse, human	[84]

		ERK1/2 MAP kinases promote cell cycle entry by rapid, kinase-independent disruption of retinoblastoma-lamin A complexes.	human	[91]

		PARP-1 usually gets activated by DNA strand breaks and is required for DNA repair. ERK2 activates PARP-1 independently of DNA strand breaks	rat	[85]

	ERK3	ERK3, an atypical member of the MAPK family, interacts with MAPK-activated protein kinase 5 (MK5 or PRAK) independent of ERK3 enzymatic activity. Erk3 regulates MK5 cellular localization and activation thus being involved in embryonic development.	mouse	[248]

	ERK5/Mpk1	Erk5, a member of the MAPK family, associates with the Paf1 complex thereby blocking Sen1-mediated premature transcription termination.	yeast, human	[249]

	Erk8	Erk8 negatively regulates transcriptional co-activation of androgen receptor and GRalpha by Hic-5 in a kinase-independent manner		[250]

	p38 MAPK	p38 inhibits cell cycle progression in a kinase-independent fashion, whereas promotes G2/M checkpoint in a kinase-dependent manner	Several human and murine cell lines	[98]

		p38 blocks transcription in proliferating cells by sequestering transcription co-activator Mirk/Dyrk1B	NIH3T3/human cell lines	[99]

	Cdc2	Cdc2 blocks cell cycle in S phase via inhibition of E2F	Drosophila	[251]

	Cdk1/cdc28	Cdk1/cdc28 recruits proteosomes to coding region to maintain transcriptional activity	yeast	[252]

	Cdk5	Cdk5 is a cell cycle suppressor in normal post-mitotic neurons	Mouse primary neurons	[253]

**CK1**	Casein Kinase 1ε	Casein Kinase 1ε regulates Fz/planar cell polarity (PCP) pathway in Drosophila development in a kinase-independent manner, whereas Wnt-Frizzled (Fz)/beta-catenin pathway requires its kinase activity	Drosophila	[254]

	VRK-3	Vaccinia-related kinase 3 (VRK-3), a member of the VRK family, suppresses ERK activity through direct binding to the MAPK phosphatase Vaccinia H1-related (VHR). VHR is known to dephosphorylate and inactivate ERK in the nucleus.		[255]

**CAMK**	AMPK	AMP-activated protein kinase α (AMPKα) acts as transcriptional co-activator of PPAR under ATP deprivation	Rat hepatocytes	[256]

	MARK2	MARK2 regulates neuronal morphology independently of kinase activity	Neuronal cells	[257]

	DAPK	Death-associated protein kinase (DAPK) increases glycolytic rate through binding and activation of pyruvate kinase		[258]

**STE**	MEK5	A constitutively active form of MEK5 is able to inhibit SUMOylation of the atypical MAPK ERK5 independent of kinase activity, but dependent on MEK5-ERK5 association	mouse	[259]

	MEKK1	The PHD domain of MEKK1 acts as an E3 ubiquitin ligase and mediates ubiquitination and degradation of ERK1/2	human	[260]

	PAK1 (p21-activated kinase1)	Recruits Akt to the plasma membrane and facilitates Akt1 and PDK1 interaction	Cell lines including Cos and NIH 3T3	[136]

		Overexpressed PAK1 induced lamellipodia formation and membrane ruffling independently of its catalytic activity	REF52 cell line	[133]

		PAK1 targeted to the plasma membrane promotes cell differentiation in the PC12 model independently of its kinase activity	PC12	[132]

		Overexpressed PAK promotes F-actin accumulation in a kinase independent manner, whereas its effect on cell shape is kinase-dependent	Swiss 3T3	[134]

		PAK1 promotes formation of multiprotein complex in focal adhesions that consists of PAK-PIX-PKL-Paxillin. Conformational change, but not kinase activity of PAK1 is required for complex formation	CHO cells	[135]

		PAK1 induces activation of exchange factor, PIX, upon binding G_βγ_. This leads to Cdc42 activation and subsequently PAK1 kinase activation.	Myeloid cells	[137]

	PAK2	PAK2 controls spindle orientation independently of its kinase activity	HeLa cells	[139]

	PAK4	PAK4 mediates TNFα-induced cell survival by promoting recruitment of TRADD to TNFα receptor	HeLa cells	[149]

		PAK4 promotes cell survival by inhibiting caspase activation	Human (HeLa cells) and mouse (NIH3T3)	[148,261]

	ASK1 (MAP3K5)	ASK1 inhibits NF-κB-induced cell survival by perturbing TRAF6-TAK1 interaction	HEK293	[80]

		ASK1 induces a Daxx-dependent caspase-independent cell death		[78]

	MST1	MST1 serine-threonine kinase, a component of the RASSF1-LATS tumor suppressor network, binds androgen receptor (AR), but the kinase activity of MST1 is not involved in inhibition of AR.	human, mouse	[262]

**AGC**	PDK1	Interacts with Ral-GDS and induces its GEF activity of in PI3 kinase dependent manner		[182]

		Forms a multiprotein complex, required for NFkB activation upon TCR activation in T cells		[181]

	Gprk2/GRK2	G protein-coupled receptor kinase 2 (Gprk2) promotes high-level Hedgehog signaling by regulating the active state of Smo through kinase-dependent and kinase-independent mechanisms in Drosophila	Drosophila	[263]

		Kinase activity-independent regulation of the cyclin pathway by GRK2 is essential for zebrafish early development.	Zebrafish	[264]

		Interaction assays of the neurokinin-1 (NK-1) receptor with G-protein coupled receptor kinases (GRKs) reveal that GRK5 interaction with the receptor was dependent on intact kinase-activity, whereas the high affinity phase of GRK2 interaction was independent of kinase activity.		[265]

	Gprk2	G Protein-coupled Receptor Kinase 2 (Gprk2) has kinase-independent functions during Histamine H2 receptor desensitization		[266]

	MSK2	Mitogen- and stress-activated protein kinase 2 (MSK2), a member of the ribosomal S6 kinase (RSK) family, functions as an adaptor in mediating activation of PKR (double-stranded RNA (dsRNA)-activated protein kinase) independent of its catalytic activity.	human	[267]

**Atypical**	mTOR (FRAP)	Differentiation of myoblasts can be rescued by Rapamycin-insensitive or Rapamycin-insensitive kinase-dead mTOR	mouse	[192]

		Dystrophin expression muscle cells	mouse	[195]

		Negative regulation of microRNA-125b expression in skeletal muscles during differentiation and muscle regeneration	Mouse	[268]

	Rad3 (ATR)	Rad3 (ATR)-Rad26 (ATRIP) complex can recruit Tel1(ATM) to telomeres independently of Rad3(ATR) kinase activity.	Fission yeast (Schizosaccharomyces pombe)	[269]

	Tel1	ATM-related protein, Tel1, regulates telomere maintenance in yeast.	Budding yeast (Saccharomyces cerevisiae)	[270]

	H11	H11 has two functions in cardiac cells: At low doses, it induces hypertrophy through kinase-independent activation of Akt, whereas at high doses H11 causes apoptosis through protein kinase-dependent mechanisms by inhibition of CK2.	mouse	[271]

**Other kinases**	Wnk	With-no-lysine (K) kinases (Wnk) regulate ion transport via both catalytic and non-catalytic mechanisms. While regulation of cation-chloride-coupled cotransporters, Na+-K+-2Cl(-) cotransporter (NKCC) 1, and NKCC2 by WNKs requires kinase activity, intersectin-mediated endocytosis of ROMK1 is independent of Wnk kinase activity.		[272]

		Wnk1 mediates activation of SGK1 downstream to the Insulin-like growth factor 1.		[200]

	WNK2	Tumor suppressor as indicated by preventing colony formation in glioma cells		[273]

	ILK	ILK interaction with α-parvin but not its kinase activity is required for embryonic development	Mouse	[274]

		ILK links the cytoskeleton and the plasma membrane at sites of integrin-mediated adhesion.	Dosphila/C. Elegans	[275,276]

		ILK regulates actin reorganization in chondrocytes and modulates chondrocyte growth independently of phosphorylation of Pkb/Akt and GSK3-beta.	mouse	[277]

		ILK regulates cell polarization, adhesion and actin accumulation at the integrin-adhesion sites	mouse	[278]

		ILK controls epidermis and hair follicle morphogenesis by modulating integrin-mediated adhesion, actin reorganization, and plasma membrane dynamics in keratinocytes	mouse	[279]

	IKKα	IKKα controls epidermis formation via regulation of keratinocyte differentiation in a NF-κB-independent fashion	mouse	[280]

	IKKβ	Regulates vascular permeability and migration of endothelial cells by regulating Akt activation.	Endothelial cells	[281]

	Aurora A/AIR-1	Aurora (AIR-1) stabilizes spindle microtubules independently of its kinase activity, however kinase activity is required for centrosome regulation	C. elegans	[282]

	TLK-1	Tousled-like kinase (TLK-1) mediates activation of Aurora B kinases independently of kinase activity thus regulating cell division	Budding yeast (Saccharomyces cerevisiae)	[283]

	Fa2p	Fa2p, a member of the NIMA-family of kinases (Neks), regulates cell cycle by associating with the proximal end of centrioles. While this cell cycle function of Fa2p is kinase independent, its function of coordinating of cilia is kinase dependent.	*Chlamydomonas, Kidney cells*	[284]

	Nek2B	Nek2B, NIMA-related protein kinase, promotes assembly of a functional zygotic centrosome independently of its kinase activity	Xenopus laevis	[285]

	Apg1/Atg1	In autophagic cells, Apg1 (Ulk-1 in human) kinase activity is required only for Cvt trafficking of aminopeptidase I but not for import via autophagy.	yeast	[286]

**Lipid kinases**	p110β	The catalytic activity of p110β is dispensable for embryonic development	Mouse	[158]

		DNA replication during the S phase		[162]

		Mediates double-strand DNA break repair via catalytic and non-catalytic mechanisms	MEF and NIH 3T3	[161]

		Endocytosis and oncogenic transformation as indicated by transferin uptake and foci formation, respectively.	MEF	[159]

	p110γ	p110γ regulates integrin activation in platelets and thrombus formation in a kinase-independent manner; p110β contributes to the same process by PIP3 production	Mouse	[287]

		Negatively regulates cardiac contractility by mediating phosphodiesterase 3B activation and thus leading to cAMP destruction	Heart (mouse)	[174]

		p110gamma binds phosphodiesterase 3B, whereas the regulatory subunit of PI3K, p87 binds Protein Kinase A (PKA). PKA, activated by cAMP phosphorylates phosphodiesterase 3B and therefore leads to negative regulation of cAMP levels in cardiomyocytes.		[176]

		Protective role during myocardial ischemia and reperfusion injury	Endothelial progenitor cells (mouse)	[169]

		Reparative neovascularisation after unilateral limb ischemia	mouse	[168]

	p85	Positivey regulates JNK activation in a response to insulin stimulation	brown adipose cells	[288]

		GTPase activity towards Rab4 and Rab5 small G proteins, as part of negative PDGF regulation. Prevents cell transformation		[289]

		p85 controls mammalian cytokinesis by regulating Cdc42 activation		[290]

	IP3K-A	IP3K-A (Ins(1,4,5)P(3) 3-kinase-A) regulates cytoskeletal organization in a kinase-independent manner	Lung epithelial cells (H1299)	[291]

**AGC **- Containing PKA, PKG, PKC families; **CAMK **- Calcium/calmodulin-dependent protein kinase; **CK1 **- Casein kinase 1; **CMGC **- Containing CDK, MAPK, GSK3, CLK families; **STE **- Homologs of yeast Sterile 7, Sterile 11, Sterile 20 kinases; **TK **- Tyrosine kinase (including receptor tyrosine kinases and non-receptor tyrosine kinases); **TKL **- Tyrosine kinase-like. (adapted from [2])

### Receptor Tyrosine Kinases (RTK)

Receptor Tyrosine Kinases (RTK) comprise a family of about 60 cell surface receptors, which act as docking platforms for polypeptide-based growth factors, cytokines, and hormones [8]. RTKs are starting points for several signalling pathways, and hence are not only key regulators of many normal cellular processes, but also play a major role in development and progression of many malignancies [9]. Among the 60 receptors of the RTK family, several family members are involved in mechanisms where no kinase activity is required.

#### The Epidermal Growth Factor Receptor (EGFR/ERBB1) ERBB family

The ERBB family of RTKs is one of the best known and most extensively studied signal transduction networks with implications ranging from cell division to cell death and motility to cellular adhesion (extensively reviewed in [10,11]). The receptor family consists of 4 members: ERBB1 (also known as HER1 or EGFR), ERBB2 (also known as HER2/neu), ERBB3 (HER3), and ERBB4 (HER4), which can form homo- and heterodimers with specific functions. 11 specific ligands are known to bind and activate ERBB receptors, which are the starting points of downstream signalling pathways such as MAPK, AKT and JNK signalling cascades [12].

The EGFR is activated by binding of several ligands, including epidermal growth factor (EGF) and transforming growth factor alpha (TGFα). EGFR forms homodimers as well as three functional heterodimers with the other members of the ERBB family, which stimulates its intrinsic intracellular protein-tyrosine kinase activity and results in autophosphorylation of several tyrosine residues in the C-terminal domain of EGFR [13]. These phospho-tyrosines serve as docking sites for an array of signal transducers [14], including kinases, phosphatases, transcription factors, and several adaptor proteins such as GRB2 and Shc, which are responsible for the initiation of multiple downstream signalling pathways. With this functional repertoire, it comes as no surprise, that deregulation of expression levels, gene amplifications and mutations of the EGFR or family members are found in ~ 30% of all epithelial cancers.

Interestingly, in cancer tissues the expression level of EGFR is correlated with prognosis, but not with responsiveness to EGFR inhibitor treatment [15]. This conundrum suggests that EGFR might contribute to tumor progression independently of its kinase activity. Several studies support this kinase-independent pro-survival function of the EGFR.

First, loss of the kinase activity of the EGFR does not produce phenotypes similar to the ablation of EGFR protein expression [16]. While EGFR knockout animals die soon after birth [17], animals expressing kinase-defective EGFR are viable and display only some epithelial defects [18].

Second, several groups reported the surprising results, that a kinase-defective EGFR was capable to activate downstream signalling (such as MAPK) and stimulate DNA synthesis, while failing to induce the tyrosine phosphorylation of endogenous substrates in response to EGF [19-22]. These kinase activity independent signalling included transcriptional effects, as kinase-deficient EGFR could activate c-fos expression [23]. The mechanism may include heterodimerization with other ERBB members. For instance, the co-expression of a kinase-inactive mutant of EGFR (K721M) with ERBB2 resulted in EGF-dependent Akt and MAPK activation., while kinase-inactive EGFR alone was ineffective [24]. ERBB2's kinase activity, but not tyrosine phosphorylation, was required for this activation. These results suggest that EGFR has catalytic-independent functions, which might be achieved by heterodimerization with other members of the ERBB receptor family.

**Figure 2:**
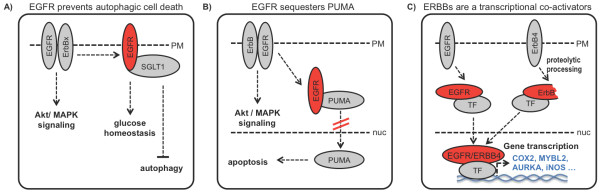
**Kinase actitvity-independent functions of the EGFR/ERBBs**. **(A) **EGFR prevents autophagic cell death by stabilizing the sodium/glucose cotransporter (SGLT1) thus maintaining the basal intracellular glucose level. **(B) **In glioblastomas, EGFR and EGFR vIII sequester the proapoptotic Bcl-2 family member PUMA in the cytoplasm leading to tumour drug resistance. **(C) **EGFR and ERBB4 regulate gene expression by direct interaction with transcription factors in the nucleus.

More recent studies provide more mechanistic insights as to the nature of kinase-independent signalling, which relies on protein-protein interactions. In 2008, Weihua et al. reported that EGFR, independently of its kinase activity, prevents cancer cells from autophagic cell death by maintaining the basal intracellular glucose level [16]. EGFR interacts with and stabilizes the sodium/glucose cotransporter (SGLT1) in order to promote glucose uptake into cancer cells (Figure 2A). Interestingly, inhibition of the EGFR kinase activity did not block this association with SGLT1 or decrease basal intracellular glucose levels suggesting that no kinase activity is required for this regulation. Similarly, the EGFR and its constitutively activated variant EGFRvIII were shown to bind to and sequester the proapoptotic Bcl-2 family member PUMA in the cytoplasm leading to tumour drug resistance [25] (Figure 2B).

These results have important implications for therapeutic approaches relying on agents that inhibit the EGFR kinase activity, as the kinase independent functions of EGFR may open escape routes, which for instance maintain the viability of tumor cells even in the presence of EGFR kinase inhibitors.

In addition to non-catalytic functions regulating downstream effectors by the EGFR via direct protein interactions, they also contribute to the regulation of the localisation of the EGFR family itself. EGFR and other ERBB receptors are known to be regulated by endocytosis. Ligand binding induces the internalization of the receptor into endosomes, where the receptors are either targeted for ubiquitin-mediated degradation or recycled back to the plasma membrane. While this process was thought to require kinase activation, newer data suggest that rather than EGFR kinase activity, dimerization is necessary and sufficient for internalization [26].

ERBB family receptors contain nuclear localisation signals [27] enabling them to translocate to the nucleus, either as full length molecules (e.g. the EGFR) or as fragments (e.g. ERBB4) after proteolytic processing [28] (Figure 2C). Full-length EGFR trafficking from the plasma membrane seems to involve the Sec61 translocon [29], where upon addition of EGF the cell surface EGFR is trafficked to the endoplasmic reticulum. There, the EGFR associates with Sec61β, and is subsequently retrotranslocated from the ER to the cytoplasm. The EGFR lacks a DNA binding domain, but can interact with several transcription factors, such as STATs3/5 and E2F1, to activate the expression of iNOS, COX-2, MYBL2 (B-Myb), and AURKA (Aurora A) genes [28]. As the induction of target gene expression could be inhibited by EGFR inhibitor drugs, gene transactivation seems to require catalytic EGFR activity. However, it is unclear whether kinase activity is needed for nuclear translocation or the actual transcriptional transactivation function of the nuclear EGFR. Interestingly, a C-terminal ERBB4 fragment lacking the kinase domain is able to activate transcription by associating with the YAP2 transcription factor [30], suggesting that the transactivation function may be independent of catalytic activity. Along these lines it has been suggested that the proteolytic ERBB4 fragment serves as a chaperone for STAT5 [31] and YAP [32] that facilitates nuclear entry of these transcription factors.

Kinase independent functions have been mainly described for the ERBB family, but may be more widespread in RTK signaling. This is not surprising given that the function of RTKs relies heavily on their abilities to assemble multi-protein signaling complexes. Although the focus has been on proteins recruited to tyrosine phosphorylation dependent docking sites, there is increasing evidence that a great number of proteins are associated with RTKs independently of ligand, and that at least some of these proteins also participate in the regulation of signaling [33].

#### Insulin like Growth Factor-1 Receptor (IGFR)

Another recent example for kinase independent signaling is the IGFR, which could stimulate the ERK pathway despite having its kinase activity blocked by chemical IGFR inhibitors or even abolished by mutation [34]. ERK activation also was independent of PI3K kinase activity or phosphorylation of IRS, which is a multivalent adaptor protein that mediates many of IGFR downstream signaling events. By contrast, ERK activation was blocked by chemical inhibition of Src family kinases or Gβ/γ subunits of heterodimeric G-proteins, indicating that G-protein coupled receptor signaling participates in the kinase independent IGFR activation of ERK.

### Kinase independent functions of Mitogen Activated Protein Kinase (MAPK) pathway components

**Figure 3:**
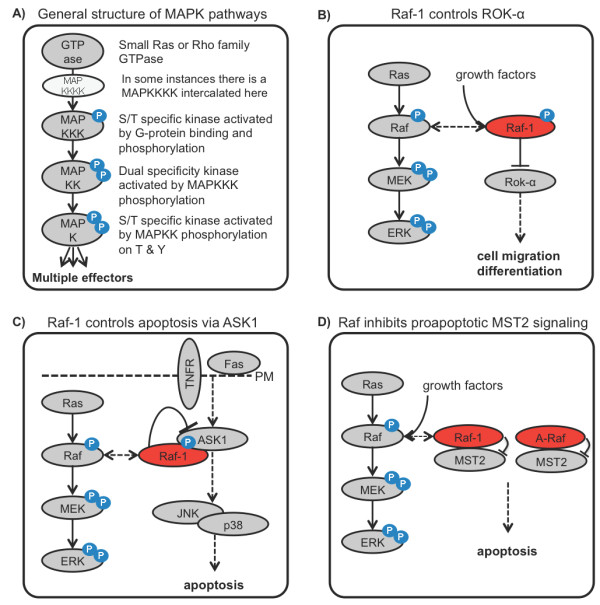
**Kinase-independent functions of Raf kinases**. **(A) **General structure of MAPK pathways. **(B) **Raf-1 controls cell migration and differentiation by inhibiting the Rho effector kinase ROK-α. **(C) **Raf-1 controls TNF- and Fas-mediated apoptosis by inhibiting apoptosis signal-regulating kinase-1 (ASK). **(D) **Raf-1 and A-Raf bind and inhibit the pro-apoptotic mammalian sterile 20-like kinase (MST2) thereby interfering with its dimerization, autophosphorylation, and activation.

MAPK pathways are ubiquitous signaling modules consisting of a three-tiered, and sometimes four-tiered, cascade of kinases that is activated by a small G-protein as input (Figure 3A). The name mitogen activated protein kinase is historic, but now indicates a range of pathways that respond to a variety of stimuli including mitogens, hormones, and stress signals [35]. The activation of the first kinase, MAPKKK, is initiated by it binding to an activated Ras or Rho family protein. MAPKKK then phosphorylates and activates MAPKK, which are dual specificity kinases that activate MAPK by phosphorylation of a tyrosine and threonine in the activation loop. While signaling within the cascade is largely linear, the terminal MAPK usually has a large number of substrates, whose phosphorylation kinetics and localization contribute to the generation of specific biological outputs [35,36]. The design of MAPK modules conveys interesting intrinsic properties, such as switchlike responses and output stabilization [37,38].

#### Kinase independent functions of Raf kinases

Raf is the MAPKKK in the first MAPK pathway identified, the Ras-Raf-MEK-ERK pathway. This cascade is a main effector pathway of ERBB receptors, and altered in a high percentage of cancers usually be mutation of Ras or BRAFgenes. ERK features more than 150 substrates thereby regulating many fundamental cellular functions, including proliferation, differentiation, transformation, apoptosis and metabolism [39].

Raf proteins come in three isoforms (A-Raf, Raf-1, B-Raf) encoded by different genes. A wealth of experimental data suggests that MEK1 and MEK2 are the only *bona fide *Raf substrates. B-Raf has the strongest kinase activity towards MEK, while Raf-1 is weaker and A-Raf activity is barely detectable [40]. From an evolutionary point of view and phylogenetic comparisons, the single Raf homologs in invertebrates (lin-45 in *Caenorhabditis elegans *and D-Raf in *Drosophila melanogaster*) are much closer related to B-Raf in terms of sequence, than Raf-1 and A-Raf [41,42]. This suggests that B-Raf is the archetypal MEK kinase, whereas A-Raf and Raf-1 may have evolved towards MEK-independent functions. Furthermore, gene ablation experiments in mice showed that Raf-1 is required for survival and protects against apoptosis [43,44]. Of note, reconstituting *Raf-1^-/-^*mice with a non-activatable Raf-1 mutant with reduced kinase activity fully rescued the apoptotic phenotype and produced viable mice [43]. Taken together, these results suggested, that Raf-1 (and A-Raf) might possess kinase-independent functions. During the hunt for new Raf targets, new kinase-independent roles for Raf proteins besides the MEK substrate have emerged. These include the regulation of cell motility and differentiation by controlling the activity of ROK-α and the regulation of apoptosis by suppressing the activity of the proapoptotic kinases ASK1 and MST2, none of which requires Raf-1 kinase activity (reviewed in [45-47]).

##### Raf heteromers

Over the years it emerged that Raf proteins are able to homo- and heterodimerize with each other [48-50]. Interestingly, these allosteric mechanisms of dimerization regulate kinase activity of the complexes. One intriguing phenomenon was that even a catalytically compromised B-Raf was capable of inducing kinase activity of Raf-1 *in trans *in a manner dependent on a physical interaction between B-Raf and Raf-1, suggesting that the underlying mechanism is independent of a simple transautophosphorylation route [46,51-53]. Only recently, the exact mechanism of how these dimers are regulated was discovered [54], which suggests that two Raf proteins are found in a 'side-to-side' dimer configuration. Several proteins seem to be responsible for the correct configuration, which include the scaffold KSR and 14-3-3 proteins [54]. Additionally, two kinases, MLK3 and DGKη can enhance and maintain Raf-1/B-Raf heterodimer formation independently of their kinase activity [55,56]. What has not been solved yet is the mechanism how a kinase dead Raf protein can stimulate the activity of another Raf protein in the context of a heterodimer, but an allosteric mechanism is the most plausible possibility. The observation that Raf-1 activation by heterodimerization with B-Raf seems to proceed differently from the activation used by growth factors [51], is in keeping with such an alternative mechanism of Raf activation exerted by allosteric changes. Interestingly, Raf-1 and B-Raf also can induce allosteric conformation changes in KSR, which conveys KSR the ability to phosphorylate MEK [57,58]. KSR is scaffolding protein that binds Raf, MEK and ERK, but whether KSR also has kinase function is controversial with most evidence indicating that at least mammalian KSR proteins lack kinase activity [59]. Although, *in vitro *MEK phosphorylation by KSR is weak and mainly occurs on sites different from the known activating sites [57], work with KSR1 knockout cells has suggested that KSR1 is required for efficient ERK pathway activation in cells [58]. This enhancement may reflect allosteric cooperativity between Raf and KSR, and maybe other MEK kinases, when assembled into multi-protein complexes in cells. This kind of complex formation may also play a pathophysiological role in cancer. It could explain the surprising finding that a small number of B-Raf mutations occurring in tumors have reduced kinase activity, and exert their oncogenic action by stimulating Raf-1 [52]. A therapeutically even more important observation is that Raf inhibitory drugs can activate the ERK pathway, and in clinical trials may be responsible for some adverse side effects of otherwise highly efficacious Raf inhibitors [60,61]. This paradoxical activation of ERK occurs in tumor cells with Ras mutations, which cooperate with Raf inhibitors to induce B-Raf-Raf-1 heterodimerization [62]. As the Raf heterodimer activates MEK at least 30-fold stronger than B-Raf, but only requires one Raf partner to have kinase activity [53], even a slightly incomplete inhibition of Raf will promote ERK pathway activation by Raf heterodimers.

##### ROK-α

ROK-α is a direct effector of the Rho GTPase, which is activated by binding to active Rho [63]. In *Raf-1 *knockout cells ROK-α is hyperactivated and mislocalized to the membrane [64,65]. Furthermore, Raf-1-mediated inhibition of ROK-α promotes cell migration and reduces sensitivity to Fas-induced apoptosis [64,65]. Stimulation with growth factors induces an interaction between the regulatory region of Raf-1 and the kinase domain of ROK-α, resulting in inhibition of ROK-α kinase activity [66] (Figure 3B). This *in trans *regulation of a kinase domain by the regulatory domain of another kinase introduces a new concept of kinase regulation that may have important implications for signal coordination where the activation of one pathway automatically would inhibit another pathway. In a more recent study, Raf-1-mediated inhibition of ROK-α was shown to block keratinocyte differentiation, thus allowing both the development and maintenance of Ras-driven epidermal tumors in mice [47,67]. Of interest, the regulation of ROK-α by Raf-1 is exclusively mediated by protein-protein interactions and does not require Raf-1 kinase activity.

##### MST2

The other proapoptotic kinase, which is inhibited by Raf-1, is mammalian sterile 20-like kinase, MST2, which was identified in a proteomics screen of Raf-1-associated proteins [68]. MST2 is activated by dimerization and autophosphorylation of the activation loop. Raf-1 interferes with MST2 dimerization and autophosphorylation by binding to the MST2 SARAH domain, which mediates MST2 dimerization (Figure 3D). Raf-1 kinase activity is not required for this regulation as kinase-dead Raf-1 mutants, or even the mutant lacking the complete kinase domain, also can inhibit MST2 activation. As a result, MST2 activity is constitutively elevated in Raf-1 knockout cells and hyperactivatable by Fas stimulation or expression of RASSF1A [68,69]. Interestingly, the Raf-1-MST2 interaction is induced by stress and relieved by mitogens. Upon stimulation of cells, Ras binding to Raf-1 enables Raf-1 to activate the ERK pathway and promote proliferation, but at the same time dissociates the MST2-Raf-1 complex and promotes apoptosis [70]. Coupling cell proliferation to the risk of cell death seems paradoxical at first sight, but this dual role makes perfect sense for higher organisms where the uncontrolled proliferation of cells can lead to severe diseases including cancer. In a subsequent study, we could show that A-Raf, the third member of the Raf family, also binds to and inhibits MST2 [71,72]. Similar to Raf-1, A-Raf kinase activity is not required as kinase-dead A-Raf mutants also can inhibit MST2 activation. However, while Raf-1 binding to MST2 is induced by stress and reduced by mitogens, A-Raf binds to MST2 constitutively and seems to promote the survival of cancer cells by restraining MST2 mediated apoptosis [71]. B-Raf binds MST2 only very weakly [68]. Thus, the observed differential MST2 binding pattern inversely correlates with the kinase activity towards MEK and the evolution of the Raf family. B-Raf as the oldest member possesses the strongest MEK kinase activity and the lowest affinity for MST2, while the youngest member, A-Raf, has the poorest MEK kinase activity but the strongest capacity to bind and inhibit MST2 [72]. This observation suggests that during evolution the role of Raf might have shifted from activating the ERK pathway to inhibiting the MST2 pathway.

From a therapeutic point of view, targeting these catalytic-independent Raf interactions (ROK-alpha, ASK1, MST2) in cancer might prove to be a good strategy. Disruption of Raf-1/ROK-alpha might provoke the differentiation of epidermal skin tumor cells, while the dissociation of Raf-1/A-Raf from ASK1 and MST2 should activate these kinases to cause apoptosis in tumor cells.

##### ASK1

Another kinase, which is regulated by Raf-1 independent of its catalytic activity is apoptosis signal-regulating kinase-1 (ASK) (Figure 3C). ASK1 works as a MAPKKK in the JNK and p38 MAPKs pathways to promote apoptosis induced by stress or by death receptors, such as the TNF- receptor or Fas [73]. Raf-1 binds to ASK1 inhibiting its kinase activity and apoptosis. Raf-1 catalytic activity was not required for the control of ASK1 as a kinase dead Raf-1 mutant inhibited ASK1 as potent as wildtype Raf-1 [74]. Although the direct mechanism of inhibition is not known yet, the pathophysiological relevance of ASK1 inhibition by Raf-1 was demonstrated in a mouse model of heart disease [75]. A tissue-specific knockout of the *Raf-1 *gene in the heart muscle resulted in ventricular dilation and fibrosis caused by an increase in cardiomyocyte apoptosis. These pathological changes could be prevented by also knocking out the *ASK1 *gene.

Interestingly, ASK1 is able to induce apoptosis in a kinase activity dependent and independent manner. The kinase dependent way executes apoptosis via the activation of JNK and subsequent inactivation of Bcl-2 [76] and stabilization c-Myc [77]. The kinase independent pathway induces a caspase-independent form of cell death, which is enhanced by the binding of ASK1 to Daxx [78]. The contribution of this kinase independent pathway to ASK1 induced cell death still needs to be clarified, as it relied on the overexpression of ASK1 or kinase defective mutants. In addition, Daxx is an activator of the ASK1 kinase activity, and Daxx induced apoptosis was reported to be blocked by a kinase deficient ASK1 mutant [79]. Another potential target of kinase-independent ASK1-induced apoptosis is the transcription factor nuclear factor-kappaB (NFB), a key regulator of immune and inflammatory responses that exerts anti-apoptotic roles in various cells. ASK1 directly interacts with TAK1 (**t**ransforming growth factor-beta-**a**ctivated **k**inase 1), a kinase involved in NFB activation in response to inflammatory and cytokine signalling. ASK1inhibits NFB activation by interfering with the formation of the TRAF6/TAK1 complex that mediates interleukin-1 induced TAK1 activation [80]. This disruption renders cells vulnerable to apoptosis upon inflammatory stress.

### ERK MAPK

ERK1 and ERK2 are the MAPKs at the end of this pathway featuring more than 150 known substrates, and it is still far from clear how these diverse functions are coordinated in order to achieve the intended biological outcome and specificity [39]. Besides their canonical kinase dependent functions, both ERK kinases were shown to influence their substrates not only by phosphorylation, but also by direct protein-protein interactions independently of ERK kinase activity [81-83]. There are only few examples, but as ERK tends to associate with its substrates in rather stable pre-activation complexes [36] this type of regulation may be more widespread than currently appreciated.

One example is topoisomerase IIa, an enzyme involved in winding and unwinding of DNA and therefore crucial in replication and transcription. Shapiro and co-workers reported that topoisomerase IIa is activated by ERK by a phosphorylation-independent process [84]. The exact mechanism of this activation is not clear. Interestingly, it requires a double phosphorylated ERK protein that is in the activated conformation, but it does not rely on ERK kinase activity itself. ERK is likely to induce a conformational change of the topoisomerase by a direct interaction, thus leading to the expected regular DNA unwinding activity of the topoisomerase [84].

**Figure 4:**
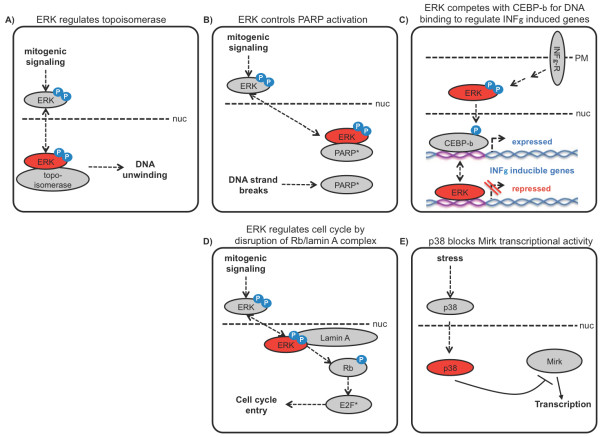
**Catalytic-independent functions of MAPKs**. **(A) **Topoisomerase IIa, involved in winding and unwinding of DNA, is activated by ERK by a phosphorylation-independent process. **(B) **Activated ERK2 interacts with PolyADP-ribose polymerase 1 (PARP-1) and activates it independent of ERK kinase activity and DNA strand breaks. **(C) **ERK can repress INFγ induced genes by directly binding to a specific DNA sequence and displacing the CEBP-β transcription factor. **(D) **Activated ERK1 and ERK2 regulate the cell cycle entry by dislodging Rb from its interaction with lamin A. Rb is released to the nucleoplasm and is rapidly phosphorylated and inactivated, leading to activation of the transcription factor E2F and cell cycle entry. **(E) **p38 inhibits cell cycle progression by blocking Mirk/Dyrk1B transcriptional activity in proliferating cells.

Another interesting kinase independent target of ERK2 is PolyADP-ribose polymerase 1 (PARP-1), where no kinase activity of ERK is required. PARPs catalyze the posttranslational modification of nuclear proteins by polyADP-ribosylation. Usually, the catalytic activity of PARP-1 is stimulated by DNA strand breaks, and its activation is required for initiation of DNA repair. Cohen-Armon and co-workers report an alternative mode of activation [85], where ERK2 interacts with PARP-1 and activates it independent of DNA strand breaks Figure 4B). Of note, these findings indicate that while phosphorylation of ERK is required for the interaction and activation of PARP-1, no kinase activity of ERK2 is necessary for this process.

Another example is an enzyme that dephosphorylates ERK, the phosphatase MKP3, which negatively regulates ERK activation [86]. MKP3 directly interacts with ERK through a region of the phosphatase termed kinase interaction motif (KIM). Interestingly, this interaction is independent of the phosphorylation status of ERK and its kinase activity, as phosphorylation of the activating residues of ERK does not induce a dissociation of the ERK-MKP3 complex. However, this interaction enhances the phosphatase activity of MKP3. In addition, MKP3 is a mitogen induced gene and located in the cytosol [87]. These traits indicate that the association of MKP3 with ERK also could be involved in a feedback regulation that eventually shifts ERK activity to the nucleus. Thus, MKP3 may not only function as inhibitor, but rather shape spatiotemporal gradients of ERK activation. This hypothesis needs further testing, but recent studies point to an important role of MKPs to regulate spatiotemporal aspects of ERK signaling [88].

Non-catalytic functions of ERK2 can be also linked with interferon signaling [89]. ERK2 was surprisingly identified in a large screen as a DNA-interacting protein. ERK binding to DNA was independent of kinase activity, direct, and to a specific DNA sequence, GAAAC, found in the promoters of interferon-γ (INFγ) responsive genes. This sequence motif is also bound by the C/EBP-β transcription factor, and ERK2 acted as a transcriptional repressor by competing with C/EBP-β for DNA binding (Figure 4C). In addition, kinase independent and dependent ERK functions may collaborate to form autoregulatory feedback loops. In the case of INFγ responsive genes, ERK can repress their transcription by direct DNA binding. However, when ERK becomes activated it can phosphorylate C/EBP-β which displaces ERK from the DNA and stimulates gene transcription. The increase in nuclear ERK caused by ERK activation eventually can dislodge C/EBP-β from the promoter again and terminate transcription. The transcriptional induction of MKPs, which deactivate ERK by dephosphorylation, ensures that C/EBP-β activation by ERK also ceases. This ability to regulate gene transcription by direct DNA binding highly increases the number of ERK targets.

This theme of competing for critical binding sites is reiterated in the context of cell cycle regulation by the ERK pathway. ERK kinase activity is crucial for promoting cell cycle entry by various mechanisms including the induction of cyclin D1, stabilization of c-Myc and regulation of cell cycle inhibitors such as p21^waf/cip ^and p27^kip^[90]. A kinase independent component was only recently discovered [91]. Lamin A, an integral part of the nuclear matrix and involved in the stabilization of chromatin structure and regulation of gene expression, was shown to be a mutually exclusive docking protein for ERK1/2 and the retinoblastoma (Rb) protein (Figure 4D). When ERK1/2 becomes activated and enters the nucleus, ERK1/2 dislodges Rb from its interaction with lamin A. Rb released to the nucleoplasm is rapidly phosphorylated and inactivated, leading to the activation of the transcription factor E2F and cell cycle entry. No kinase activity from either ERK1 or ERK2 was necessary for these processes [91].

### p38 MAPK

The mammalian p38 subfamily of MAPKs comprises 4 members (α, β, γ and δ) that are differentially expressed in various tissues, and activated in response to a wide range of extracellular stress stimuli, including cytokines and growth factors [92]. Numerous data obtained from mouse knockout studies and selective pharmacological inhibitors implicate an important role of p38α MAPK in inflammatory and immune responses [93-95]. More recent studies show that the mammalian p38 MAPK pathway behaves as a cell cycle inhibitor in both G1/S and G2/M checkpoint controls in response to stress stimuli [96,97]. Whereas the role of p38 in cell cycle regulation was clearly established, some of these functions may not require p38 catalytic activity. For instance, p38α depletion, but not its specific pharmacologic inhibition, impeded cell proliferation and caused mitotic arrest, revealing p38α functions independent of its kinase activity [98]. Moreover, these phenotypes were reversed by the ectopic expression of a kinase-dead p38α mutant. Finally, overexpression of wild type or kinase-negative p38α also strongly inhibited cell proliferation, proving that p38α also has a key kinase-independent function [98]. However, despite the clear absence of requirement for p38 catalytic activity to regulate cell cycle progression, the exact mechanism used to achieve this function is unknown.

Mirk/Dyrk1B protein kinase is a potential candidate to explain a kinase-independent role of p38 in cell cycle regulation (Figure 4E). Mirk functions as a transcriptional activator of genes involved in the response to certain stress agents [99]. Co-immunoprecipitation experiments demonstrated that both wild type and kinase-inactive p38 bind to Mirk and prevent its association with upstream activators and transcriptional co-factors. Interestingly, this effect is isoform specific, as only the p38α and p38β, but not the γ or δ isoforms, bind to Mirk. In addition, the observation that Mirk protein levels were variable while p38 levels stayed constant suggested that endogenous p38 could only block Mirk function when Mirk levels were low (S phase) and not when Mirk levels were elevated (G_0_/G_1_) [99]. These observations emphasized a novel cell cycle-dependent function for p38, suppressing Mirk transcriptional activity in a kinase-independent fashion only when cells are proliferating.

### Kinase independent functions in regulating cell adhesion and migration Src

Src belongs to a family of eight non-receptor tyrosine kinases and is implicated in a wide variety of signal transduction pathways. Src proteins regulate many fundamental cellular processes, such as proliferation, differentiation, cell shape, migration and apoptosis [100-102]. These pleiotropic functions of the Src family explain the importance of these kinases within the signalling machinery, and it comes as no surprise that most members of this family have been identified as oncogenes [103]. Src proteins feature a modular structure of three domains, the N-terminal SH3 domain, the central SH2 domain and a tyrosine kinase domain. The SH2 and SH3 domains cooperate in regulating the auto-inhibition of the kinase domain. Additionally, an inhibitory tyrosine phosphorylation in the C-terminus acts a binding site for the SH2 domain, which locks the protein in a closed conformation, and further facilitates binding of the SH3 domain to a linker region between the SH2 and kinase domain. This architecture allows for multiple mechanisms for c-Src activation, either by dephosphorylation of the C-terminal tyrosine by protein tyrosine phosphatases or competitive binding to the SH2 and SH3 domains by other proteins. The list of Src family kinase substrates is long [104,105], but here we briefly discuss targets of Src, which are regulated in kinase independent way.

Activation of integrin signalling is associated with a transient increase in Src kinase activity and translocation of Src to the focal adhesion complex and subsequent phosphorylation of focal adhesion kinase (FAK) [106-108]. Src-deficient fibroblasts were shown to have defects in cell spreading which is presumably related to the activation of Src by integrins. Complementation of this phenotype does not require kinase activity of Src, as cell spreading is restored by either wild type or kinase-defective Src [109]. In a similar fashion, activation of the Src-dependent adaptor protein pp130^CAS ^in fibroblasts in response to fibronectin binding does not require Src kinase activity [110]. Overexpression of Src in human colon cancer cells induces FAK phosphorylation on several tyrosine residues. Surprisingly, only one phosphorylation site requires the catalytic activity of Src, whereas phosphorylation on four other sites requires the intact SH2 domain of Src but not its catalytic activity [111]. Ablation of both Src alleles in the mouse causes osteopetrosis due to an intrinsic defect in osteoclasts [112]. Surprisingly, re-expression of kinase-defective version of *c-Src *led to a reduction in osteopetrosis in Src^-/- ^animals and partially rescued a defect in cytoskeletal organization observed in Src^-/- ^osteoclasts. These results suggest that no kinase activity is required for this phenotype.

These results suggest important kinase independent functions of Src in integrin signalling and cytoskeletal organization. This is not surprising given that they strongly rely on spatial organization, which may be better suited to be negotiated by protein interactions rather that catalytic activities.

However, while cytoskeletal organization is the prime paradigm for kinase independent Src family functions, there is also evidence for kinase independent signalling in other roles. These examples include the organisation of multiprotein signalling complexes in signalling by cytokines [113], the T-cell receptor [114], and the B-cell receptor [115]. The evidence is sparse but consistent, suggesting that we are far from having fathomed the kinase independent functions of Src family kinases.

#### Focal Adhesion Kinase (FAK)

**Figure 5:**
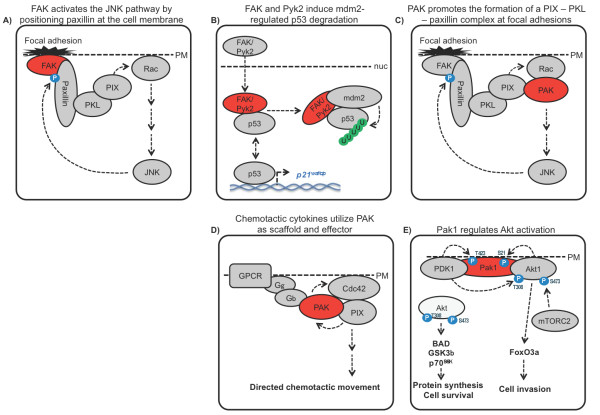
**Non-catalytic functions of FAK and PAK kinases in cell motility and survival**. **(A) **FAK mediated translocation of paxillin to the cell membrane initiates activation of the JNK pathway and cell motility by recruiting the Rac/Cdc42 exchange factor PIX. **(B) **FAK and the related kinase Pyk2 can translocate to the nucleus where they bind p53 and Mdm2 to induce p53 ubiquitination and degradation, promoting cell proliferation and survival. **(C) **PAK1 regulates focal adhesion dynamics by promoting paxillin recruitment to focal adhesions. **(D) **Chemotactic cytokines via Gβ/γ proteins induce a positive feedback activation of Cdc42 by activating Rac, which in turn activates the Rac/Cdc42 exchange factor PIX. **(E) **PAK1 scaffolds the activation of Akt1 by PDK1 at the cell membrane and coordinates the selective phosphorylation of downstream Akt1 substrates.

FAK is a tyrosine kinase mediating integrin signalling and also participating in signal transduction by growth factor receptors [116,117]. FAK contains an N-terminal FERM (band 4.1, ezrin, radixin, moesin homology), a kinase and a C-terminal FAT (focal adhesion targeting) domain. The FAT domain links FAK to integrins and focal adhesions, whereas the FERM domain connects FAK to membrane growth factor receptors and is also responsible for nuclear translocation. Early reports suggested that FAK could play a scaffolding role mediating crosstalk between signalling pathways. Indeed, FAK has been shown to induce anchorage-dependent JNK activation in a kinase-independent fashion by interacting with paxillin [118]. The exact mechanism is not known but seems to rely on FAK promoting paxillin localization to the cell membrane and the recruitment of paxillin kinase linker (PKL) and PAK-Interacting exchange Factor (PIX) [119], a guanine nucleotide-exchange factor (GEF) that activates Rac, which in turn activates the JNK pathway [120,121] (Figure 5A). Interestingly, JNK phosphorylation of S178 in paxillin enhances the binding of FAK to paxillin [122] creating a positive feedback loop that could promote the accumulation of paxillin at focal adhesions.

Importantly, FAK plays a crucial role in vascular development, and mice lacking FAK in their vascular system die before birth due to defects in angiogenesis and apoptosis of endothelial cells [123]. Mice expressing a kinase-deficient FAK mutant selectively in endothelial cells still died before birth, but the embryos survived longer than animals without any FAK in their vascular system [124]. Expression of kinase-dead FAK did not correct the vessel malformation, but enhanced endothelial cell survival by a mechanism involving the downregulation of the cyclin-dependent kinase inhibitor p21^waf/cip^[124]. p21^waf/cip ^was one of the first p53 target genes identified [125]. FAK inactivation during mouse development results in p53- and p21-dependent mesodermal cell growth arrest. By creating FAK^-/-^p21^-/- ^double knockout fibroblasts Lim et al. [126] showed that FAK, in a kinase-independent fashion, causes p53 instability via enhanced Mdm2-dependent p53 ubiquitination (Figure 5B). p53 inactivation required the FAK FERM domain for nuclear localization, p53 binding and connections to Mdm2, the p53 specific ubiquitin ligase responsible of p53 degradation. These observations defined a scaffolding role for nuclear FAK in facilitating cell survival through enhanced p53 degradation [126].

Pyk2 is a cytoplasmic tyrosine kinase related to FAK and sharing similar domain structure. Interestingly, compensatory Pyk2 expression was observed in FAK^-/- ^mice [127], suggesting some redundancy between FAK and Pyk2 functions. Indeed, FAK knockdown induced p53 activation and cell cycle arrest in endothelial cells, but a subsequent increase in Pyk2 expression led to p53 downregulation and the release of cell cycle block [128]. Moreover, in FAK^-/-^p21^-/- ^double knockout mouse embryo fibroblasts, an increase of p53 levels associated with inhibition of cell proliferation was observed only when Pyk2 was downregulated [128]. Thus, similar as FAK, Pyk2 promotes Mdm2-dependent p53 ubiquitination to facilitate cell proliferation and survival in a kinase-independent manner (Figure 5B).

#### PAK

p21-activated kinase 1 (PAK1) is a serine/threonine kinase belonging to the highly homologous group I of PAKs (PAKs 1-3). PAK1-3 activation is mediated by Rho family small G proteins, including Rac and Cdc42, which bind PAKs and induce its conformational change leading to exposure and activation of the catalytic domain [129]. PAKs are associated with a wide variety is cellular functions, including regulation of the MAPK pathway, cytoskeletal regulation and motility, differentiation, carcinogenesis and tumor invasion [129-131]. Several early studies demonstrated that PAKs have some kinase-independent functions including cytoskeletal regulation and differentiation [132-134]. On the molecular level, PAK was demonstrated to coordinate the formation of a multi-protein complex, which contains PAK, PIX, PKL and Paxillin in focal adhesions (Figure 5C). This activity required the conformational change of PAK, since activated Rac or Cdc42 were able to promote this effect [135]. As described above, the scaffolding function of FAK seems instrumental for recruiting paxillin and activating Rac via PKL and PIX (Figure 5B). Active Rac binds PAK, which is not only a Rac effector but also interacts with PIX, which in turn stimulates Rac. Thus, multiple protein interactions coupled to positive feedback loops that promote protein recruitment may by key to focal adhesion formation and function by forming a temporally dynamic yet physically stable platform.

While these studies referred to a scaffolding function of the PAK N-terminal regulatory domain, later studies pointed out new, kinase-independent functions of the C-terminal PAK kinase domain [136,137]. A dual, kinase-dependent and -independent role of PAK1 was demonstrated in myeloid cell migration. Chemokine binding to the G Protein Coupled Receptor (GPCR) induces a direct interaction between G_βγ _and PAK1. This interaction leads to activation of PIX and activation of Cdc42, which in turn leads to activation of PAK, an event required for the directional sensing of neutrophils [137]. The conformational change of PAK1 induced by the G_βγ _binding can prevent formation of PIX dimers, and therefore facilitate its GEF activity towards Cdc42 [137,138]. In this system, PAK1 mediates activation of its own catalytic activity, thus forming a positive feedback loop [137] (Figure 5D). In general, PAK1's ability to function as a scaffold for the GEFs that activates the GTPases, which activate PAK1, seems to generate an organising principle that enables cells to coordinate complex spatiotemporal responses. Similarly, PAK2 was recently described as regulating Cdc42-induced actin reorganization and spindle orientation by directly binding to the β-PIX GEF [139].

Another important PAK target is Akt, which has a central role in the regulation of metabolism, apoptosis, cell migration and transformation [140]. Whereas some studies suggest that PAK might directly phosphorylate Akt at the activating site S473 [141,142], a recent study demonstrates that PAK can also facilitate Akt phosphorylation in a kinase-independent manner [136] (Figure 5E). The kinase domain of PAK1 directly interacts with Akt and mediates its translocation to the plasma membrane, where S473 can be phosphorylated by mTORC2 [143] or PAK itself [141,142]. This step requires an activating conformational change of PAK induced by Rac binding, but no PAK kinase activity *per se*. PAK1 also recruits PDK1, the Akt T308 kinase [144], and this interaction is greatly facilitated by growth factors [136]. Thus, PAK1 promotes Akt translocation to the plasma membrane and serves as a scaffold that facilitates the interaction of PDK1 with Akt. Interestingly, PAK1mediated Akt activation exhibits isoform selectivity, affecting Akt1 responses more than Akt2, and in addition biased Akt substrate selection [136]. Expression of the N-terminal regulatory domain of PAK1, which seems to interfere with the ability of PAK to recruit PDK1, preferentially reduced the phosphorylation of nuclear Akt substrates, such as FoxO3a, while the phosphorylation levels of the cytosolic substrates S6K, BAD and GSK3 remained intact. This suggests that PAK scaffolding might direct the substrate specificity of Akt or limit its accessibility towards a subset of its substrates or both. It remains unclear, however, how the PAK dependent PDK1-Akt complex, which is formed at the cell membrane, affects nuclear Akt substrates. On the other hand, both Akt and PDK1 participate in PAK1 regulation. Akt can phosphorylate and partially activate PAK1 [145], and PDK1 was shown to activate PAK1 by direct phosphorylation in the activation loop [146]. Thus, kinases seem to be able to mutually activate each other in the PAK1 scaffolded PDK1-Akt complex by positive feedback loops, which is likely to result in a switchlike, digital signal output [147].

Members of the constitutively active group II PAK family (PAKs 4-6) also have been shown to mediate part of their effects via kinase-independent functions. PAK4 protects cells from apoptosis induced by death receptors in a kinase-independent manner by interfering with the recruitment and activation of caspase 8 to the death domains in the receptors [148]. In addition, PAK4 is needed for the efficient activation of survival pathways in response to TNFα by facilitating the binding of the scaffold protein TRADD to the activated TNFα receptor partly through a kinase-independent mechanism [149]. The role for PAK4 in regulating prosurvival pathways in a kinase-independent manner is a new function for this protein, and may help explaining its role in tumorigenesis [150,151] and development [152].

### Kinase independent regulation of phosphoinositide signaling pathways

#### Phosphatidylinositol 3-kinases (PI3K)

PI3Ks are intracellular lipid kinases that phosphorylate the 3'-hydroxyl group of phosphatidylinositol and phosphoinositides [153]. Their action is counteracted by the Phosphatase and tensin homolog (PTEN) phosphatase, which is frequently altered in cancer [154,155]. PI3Ks are divided into three classes according to their substrates specificity and sequence homology. Class I PI3Ks are activated by surface receptors and preferentially generate phosphatidylinositol-3,4,5-trisphosphate (PIP3) from phosphatidylinositol-4,5-bisphosphate (PI4,5P2) [153]. Class I PI3Ks are heterodimers consisting of a catalytic and a regulatory subunit. Catalytic domains p110α, p110β or p110δ form together with p85, p55 or p50 regulatory domains class IA PI3Ks. Class IκB PI3K consists of the catalytic p110γ and regulatory p101 or p84/p87 subunit [153,156]. Despite the fact that all class I PI3Ks generate PIP3, their simultaneous expression suggests that some of their functions might be not redundant. Indeed, a comprehensive study done by Knight and colleagues revealed specific functions of class PI3Ks in insulin signalling [157]. For instance, p110β is important for maintaining the PIP3 pool in unstimulated myotubes and balancing PTEN, a lipid phosphatase, activity thus setting a threshold for Akt activation. It is also required for Akt activation in a response to lysophosphatidic acid (LPA) stimulation [158-160]. However, p110β is dispensable for Akt activation induced by insulin, since p110α is the major PIP2 kinase in this signalling pathway [157]. Surprisingly, in some cases the phenotype observed after catalytic subunit knockdown was much more severe than the phenotype induced by saturating concentrations of an isoform-specific chemical inhibitor [161,162]. This suggests the possibility that catalytic subunits of PI3 kinase might fulfil other, kinase independent functions in cellular regulation. These, kinase-independent functions of PI3Ks were elegantly validated by obtaining knock-in homozygote mice bearing kinase dead mutants of PI3 kinase. Importantly, the phenotype of these knock-in mice was markedly different and less severe than the corresponding knockout phenotypes [163]. Here, we discuss some specific examples of non-catalytic PI3 kinase functions.

##### p110β

**Figure 6:**
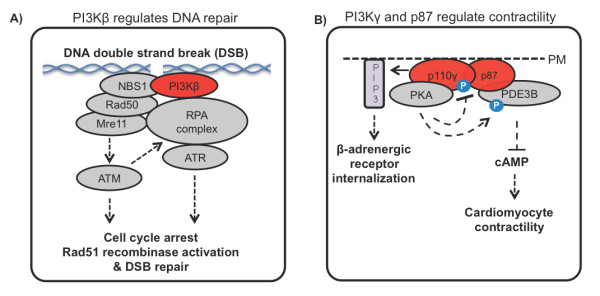
**PI3K signaling independent of catalytic activity**. **(A) **PI3Kβ binding to DNA double strand breaks (DSBs) recruits DNA damage sensor and DNA repair enzymes. **(B) **PI3Kγ comprising of the p110γ and p87, catalytic and regulatory subunits, serves as scaffold for a Protein Kinase A (PKA) and phosphodiesterase 3B (PDE3B) containing protein complex that regulates β-adrenergic receptor internalization and muscle contractility in cardiomyocytes.

p110β can be activated by growth factors and insulin. Several recent studies identified both kinase-dependent and independent roles for this isoform [157-159]. p110β is dispensable for Akt phosphorylation induced by insulin, IGF-1 (Insulin-like Growth Factor), Epidermal Growth Factor (EGF) or Platelet-derived Growth Factor (PDGF) stimulation [158]. However, the catalytic activity of p110β is required for Akt activation mediated by lysophosphatidic acid (LPA) [158,159], and a lack of catalytically active p110β protected mice from breast cancer development induced by ERBB2 or from prostate tumour development driven by loss of *PTEN *[158,159]. The latter phenomenon can be attributed to a basal catalytic activity of p110β and its balancing effect towards PTEN activity [157]. On the other hand, kinase-independent functions of p110β are crucial for several physiological processes, including embryonic development. Mice homozygotic for kinase-dead p110β develop normally and survive to adulthood, except growth retardation and developing a mild insulin resistance with age [158], whereas the p110β knockout leads to embryonic lethality in mice [164]. Further, catalytically inactive p110β facilitates endocytosis in a rate similar to the wild type p110β subunit, as indicated by transferrin and EGFR uptake [158,159].p110β, unlike p110α, can be found in the nucleus [165], and recent studies revealed kinase-dependent and independent functions of p110β in DNA replication and repair [161,162]. In response to DNA double strand breaks (DSBs) PI3Kβ participates in sensing the damage by mediating the binding of the DNA damage sensor protein Nbs1to DSBs and the recruitment of the full MRN (Mre11, Rad50, Nbs1) complex, which then activates the DNA damage kinase ATM. The assembly of this complex does not require PI3Kβ catalytic activity, although kinase competent PI3Kβ is more efficient [161]. ATM then recruits the replication protein A (RPA) complex and the ATR kinase. ATR and ATM are checkpoint kinases which halt the cell cycle until the DNA damage is repaired by the Rad51 recombinase (Figure 6A). The defective DNA repair in p110β knockout cells leads to genomic instability and chromosomal aberrations. However, cells expressing kinase-dead p110β were predominantly normal, clearly demonstrating that kinase-independent functions of p110β are sufficient to upkeep radiation-induced DNA repair [161].

In DNA replication the catalytic activity of p110β is required for the efficient progress of the replication fork [162]. p110β regulate PCNA (proliferating cell nuclear antigen) binding to the DNA and p21^waf/cip ^phosphorylation. Surprisingly, the downregulation of p110β by shRNA revealed also kinase-independent function of p110β. p110β contributes to nuclear localization of Akt independently of its catalytic activity. In addition, the ability of p110β to bind PCNA and to facilitate its chromatin loading is at least partially independent of the p110β catalytic activity [162]. This study provides us with a molecular mechanism of p110β's kinase-independent role in cell cycle previously demonstrated by BrdU incorporation assay [159].

##### p110γ

Unlike the ubiquitously expressed p110α and p110β subunits, p110γ expression is limited to certain tissues, including the hematopoietic system, endothelial cells, and cardiomyocytes [166,167].

Ischemia-induced angiogenesis is dependent on the PI3Kγ subunit. However, mice expressing a kinase-dead p110γ mutant entertain a normal reparative neovascularization, as indicated by capillary density and blood flow recovery. In detail, p110γ has a kinase-independent function in endothelial progenitor cells (EPC) migration induced by the chemokine Stromal-cell Derived Factor 1 (SDF-1), while EPC incorporation into vascular networks requires the catalytic activity of p110γ [168].

Another study described the protective role of PI3Kγ in myocardial ischemia and reperfusion (M I/R) injury. PI3Kγ knockout mice had massive immediate and long-term heart damage induced by myocardial ischemia/reperfusion. The cardioprotective role of p110γ did not require its kinase activity, since catalytically inactive p110γ knock-in mice had phenotypes similar to their wild-type littermates [169].

Of interest, PI3Kγ knockout cardiomyocytes showed a higher contractility, and PI3Kγ was shown to act as a negative regulator of contractility [170]. In cardiac hypertrophy models using chronic pressure overload caused by transverse aortic constriction (TAC) [171,172] PI3Kγ activity and expression levels were upregulated in the heart [173,174]. PI3Kγ knockout mice develop severe myocardial damage in response to TAC surgery suggesting that PI3Kγ has a protective role. Mice expressing kinase-dead PI3Kγ had preserved myocardial function a week after TAC, despite the lack of Akt and Erk activation in a response to TAC-induced mechanical stress. This can be explained by an ability of PI3Kγ to form a complex with phosphodiesterase 3B (PDE3B) independently of its kinase activity. PDE3B is associated with PI3Kγ in a constitutive manner and this interaction is required for PDE3B phosphodiesterase catalytic activity [174]. Furthermore, the PI3K regulatory subunit, p87 but not p101 undergoes a physical interaction with PDE3B [175]. Thereby, PI3Kγ helps to maintain cAMP levels and thus prevents tissue necrosis independently of its kinase activity [174].

A subsequent study revealed the molecular mechanism of how cAMP levels in cardiomyocytes are regulated by PI3K independently of the PIP3 levels [176] (Figure 6B). While the regulatory subunit of PI3K, p87, directly binds phosphodiesterase 3B (PDE3B), the catalytic subunit, p110γ, binds Protein Kinase A (PKA). cAMP, regenerated in cardiomyocytes upon Β-adrenergic receptor stimulation, leads to activation of PKA. Catalytically active PKA phosphorylates and activates PDE3B, thus providing a negative feedback regulation. In this system, PI3K serves as an anchoring protein, bringing PKA in proximity to its substrate. In addition, PKA phosphorylates p110γ and inhibits its kinase activity, thus decreasing the PIP3 levels. Since PIP3 is required for β-adrenergic receptor internalization, PKA helps to maintain receptor levels on the plasma membrane. In failing hearts, PI3K kinase activity is elevated, partially as a result of high expression levels of p110γ. In addition to that, the expression level of the regulatory subunit p101 is elevated as well. Since this subunit does not interact with PKA, the p110γ bound to p101 remains constitutively active leading to β-adrenergic receptor downregulation and heart failure [176].

#### PDK1

Phosphoinositide-Dependent Kinase 1 (PDK1) is a major regulator of at least 23 AGC kinases. Surprisingly, PDK1 is constitutively active and cell stimulation does not change its phosphorylation status at the activation loop or the kinase activity [177,178]. PDK1 mediates its own phosphorylation, thus explaining why the protein expressed in bacteria is phosphorylated and fully active; most likely this phosphorylation proceeds by a trans-molecular mechanism [178]. In the PI3K signalling pathway PDK1 participates in Akt activation. Upon PIP3 accumulation mediated by PI3Ks Akt is recruited to the plasma membrane via its PH domain binding to PIP3 or by association with PAK1 as discussed above. PDK1then phosphorylates Akt at the activation loop (T308) [177,178]. PDK1 itself contains a PH domain, however it remains controversial whether cell stimulation affects its localization within the cell [179,180].

**Figure 7:**
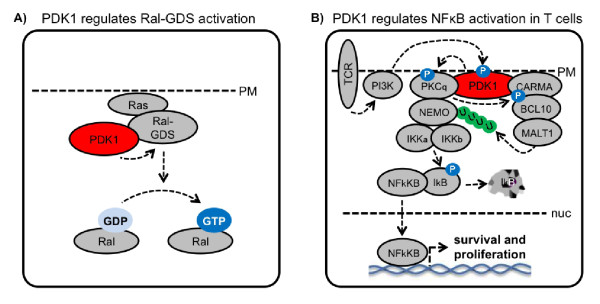
**Catalytic-independent functions of PDK1**. **(A) **PDK1 induces activation of the Ral small G protein by binding to and relieving its GEF, Ral-GDS from the autoinhibitory conformation. **(B) **PDK1 mediates NFB activation in T-cells by serving as a scaffold that brings CARMA1-BCL10-MALT1complex proximal to the PKCq-bound IKK complex, thus allowing ubiquitination of NEMO (IKKγ). This leads to activation of IKKs, phosphorylation and subsequent degradation of the NFκB inhibitor IκB and release of active NFκB into the nucleus.

Recent studies suggested that PDK1 has additional, kinase-independent functions in the coordination of signalling pathways [181,182]. For instance, PDK1 is involved in activating the small G protein Ral [182]. Ral is a small GTPase whose activation is mediated by a Ral specific GEF, Ral-GDS (Ral-Guanine nucleotide Dissociation Stimulator) [183]. Besides Raf kinases and PI3K, Ral-GDS is one of the three classic Ras effectors [184,185]. Ras contributes to Ral activation by bringing the Ral-GDS proximate to its substrate, but is not sufficient for the full catalytic activation of Ral-GDS [186]. Therefore, an additional step is required for optimal Ral activation, and PDK1 was demonstrated to fulfil this complementary role [182] (Figure 7A). PDK1 binds the N-terminal region of Ral-GDS and relieves it from inhibition. This N-terminal part of Ral-GDS was shown to possess autoinhibitory activity and Ral-GDS lacking this region has higher basal GEF activity. This function of PDK1 is kinase-independent, since a kinase-dead PDK1 mutant performed indistinguishably from wild type PDK1. Moreover, PDK1 lacking 50 amino acids in its regulatory domain did not enhance Ral-GDS activity, but was able to phosphorylate Akt at T308. These results demonstrate a clear distinction between kinase-dependent and -independent functions of PDK1 in response to growth factor stimulation [182].

Another example of PDK1 acting as a scaffolding protein was described in T-cells, where PDK1 takes part in NFκB activation upon T-cell receptor (TCR) activation. PDK1 phosphorylates and recruits PKCθ to the plasma membrane and also recruits the CARMA1- Bcl10 -MALT1 complex, bringing it proximal to the PKCθ-bound IκB kinase (IKK) complex, which consists of two kinases IKKα/β and a scaffolding protein IKKγ or NEMO. The MALT1 complex ubiquitinates NEMO leading to the activation of IKKα/β and phosphorylation of IκB. Phosphorylation targets IκB for degradation thereby releasing NFκB to the nucleus where it activates the expression of survival and proliferation genes. Thus, PDK1, in addition of being the PKCθ kinase, also serves as a nucleating factor that assembles a multi-protein complex mediating NFκB activation downstream of TCR [181]. This multi-protein complex might also facilitate the PKCθ-mediated phosphorylation of CARMA-1, which enhances the recruitment of BCL10-MALT1. This event is absolutely required for TCR-induced NFκB activation [187]. Therefore, PDK1 fulfils a dual role operating both as a kinase and as a scaffolding protein that promotes formation of a multi-protein complex required for NFκB activation in T cells.

#### Mammalian target of rapamycin (mTOR)

mTOR kinase is found in two distinct multi-protein complexes with different substrate specificity, mTOR complex 1 (mTORC1) and mTORC2, that contain Raptor or Rictor as function defining elements, respectively. mTORC1 activation is mediated by Akt downstream to PI3K, whereas rather little is known about mTORC2 regulation [188]. The ability of mTORC1 to phosphorylate its downstream targets can be negatively regulated by rapamycin. It is unclear whether rapamycin affects mTOR kinase activity or prevents mTORC1 from interacting with its substrates, since rapamycin-bound mTOR complex 1 can still phosphorylate some of its substrates or autophosphorylate under certain conditions [189-191]. Therefore, the ability of rapamycin to block a particular signaling event does not indicate that this event necessarily requires the catalytic activity of mTORC1.

Rapamycin prevented the differentiation of C2C12 mouse myoblast cells into skeletal muscle cells. This inhibition could be rescued by a rapamycin-resistant mTOR mutant [192]. Surprisingly, mTOR bearing an additional mutation that abolishes its kinase activity could also mediate cell differentiation. Of note, during cell differentiation the mTOR expression levels are upregulated on a posttranscriptional level, but no upregulation of mTOR kinase activity could be observed [192]. Another study performed in C2C12 cells demonstrated that mTOR mediated muscle cell differentiation did require its kinase activity [193]. The reason for this discrepancy is unclear.

However, subsequent studies, performed both in *in vivo *and in *in vitro *models, support the kinase independent function of mTOR in muscle cell differentiation and maintenance, and provide a molecular explanation. miRNA-125b expressed in skeletal muscle cells negatively regulates muscle differentiation and injury-induced muscle regeneration by downregulating IGF-II, which is required for this process. mTOR negatively regulates miRNA-125b expression in repairing tissue, and that this mTOR function does not require its catalytic activity [194]. The conditional knockout of mTOR in muscle cells led to severe myopathy and premature death in mice. Among other changes induced by the mTOR knockout, cells lacking mTOR, but not raptor or rictor, had decreased expression levels of dystrophin. Dystrophin expression in muscle cells is regulated by mTOR on the transcriptional level independently of its kinase activity. Chromatin immunoprecipitation (ChIP) experiments indicated that mTOR binds to the dystrophin promoter [195]. Thus, this study indicates a kinase independent, but also raptor and rictor independent functions of mTOR in transcriptional regulation [195].

### Pseudokinases

Interestingly, 48 out of the reported 518 kinases in the human genome seem to have lost their kinase activity altogether due to mutations of critical amino acids in their kinase domains thought to be required for catalytic functions. These so-called pseudokinases still have important catalysis-independent functions, e.g. as scaffolding proteins [59,196]. It is beyond the scope of this review to discuss the function of pseudokinases in detail, and we refer readers to recent reviews dedicated to this topic [46,196,197]. Within this review we only briefly discuss recent results indicating that the distinction between kinases and pseudokinases may be blurred. Pseudokinases seem to have developed from standard kinases by mutation of a few key catalytic residues while preserving the general primary and tertiary sequence organisation of the classic well-conserved kinase domains. Thus, the substrate binding properties of the kinase domains have evolved to become their main functions. This transition, however, seems to be gradual, and there is an increasing number of examples where pseudokinases can acquire kinase activity under certain conditions.

In the case of the Wnk kinase family, the general structural conservation of the kinase domain allows for a slightly modified catalytic mechanism despite lacking key catalytic residues characteristic for classic kinase domains [198-200]. Another example of a pseudokinase that uses an unconventional catalytic mechanism is the EGF receptor family member ErbB3/HER3. The traditional view was that ErbB3 lacks kinase activity but is phosphorylated and transactivated via dimerization with the active kinase ErbB2/HER2 [201,202]. Recent structural and molecular dynamics based modelling studies showed that the ErbB3 pseudokinase domain uses an alternative phosphotransfer mechanism to transautophosphorylate its intracellular region [203]. These recent results might suggest, that pseudokinases might be more active than anticipated by using alternative catalytic mechanisms.

Interestingly, in some cases these unorthodox catalytic activities may rely on very specific allosteric activation mechanisms exerted by the substrates and associated modulator proteins. A recent example is the scaffold KSR2, member of the extended Raf family and well known for its role in coordinating Raf-MEK-ERK complexes [59,204], was shown to possess catalytic activity towards MEK [205]. This activity was dependent on Raf-1 binding to KSR2 and due to an allosteric activation mechanism. The KSR2 targeted phosphorylation sites in MEK were different from the known regulatory sites suggesting that the combination of a scaffold with the allosteric activation of a pseudokinase domain may confer exquisite substrate specificity. On the other hand, the observed KSR2 mediated MEK phosphorylation was of very low stoichiometry and its physiological relevance remains to be tested.

### Technical notes

Most kinases mentioned in this review were first identified as enzymes, and only later additional functional functions were identified. Whereas the catalytic activity of a kinase is relatively easy to test, assaying for kinase-independent functions is more far more difficult. It still is challenging to determine kinase-interacting partners, and more importantly whether these protein-protein interactions contribute to the regulation of signalling independently of catalytic activity. In many studies kinase mutants lacking a kinase domain or bearing kinase-inactivating mutations were overexpressed, and the phenotype obtained was attributed to a non-catalytic activity of the protein. These studies contributed much to our current understanding of kinase activities in different systems. However, the massive overexpression of a mutated protein will change the stoichiometric ratios between interacting proteins and hence may not accurately reflect physiological phenomena. Novel technologies, such as small interfering RNA or knockin mice, allow creating a clean temporary or permanent background where the contribution of any particular kinase can be assessed without overexpression. In this case, the rescue of a phenotype with a kinase-dead kinase can serve as a much more reliable indication of non-catalytic kinase activities.

## Conclusions

One of the clear functions of kinases within signalling networks is to receive signals from an upstream regulator and to amplify it by phosphorylating multiple substrate molecules. Any function independent of catalytic activity will be governed by purely stoichiometric relationships and will be lacking the amplification potential. However, while losing the advantage of signal amplification this mechanism might offer other advantages. Therefore, it is legitimate to ask: Why do protein kinases have non-catalytic functions? There are likely many more phosphorylation sites than proteins [206,207] not only raising questions about the functional roles of these phosphorylations, but also about the specificity of protein kinases. In fact, the specificity of protein kinases seems so poor that the expansion of tyrosine kinases went hand in hand with a reduction of the number of tyrosines in proteins [208]. Thus, is it the quest for specificity rather than catalytic efficiency that rules the kinase world? The first call on specificity is the kinase domain itself and the range of substrates it can interact with. The protein kinases that essentially consist of a kinase domain only, such as MAPKs, usually feature a large number of substrates and a large number of scaffolding proteins that direct them to selected substrates. In addition, their substrates have developed dedicated docking domains [82]. In contrast, large kinases with a complex domain structure, such as the PI3Ks are often remarkably specific, and usually function as scaffolds themselves. A crucial finding was the existence of SH2 and SH3 interaction domains by Tony Pawson's group. Many kinases described above contain multiple such protein-protein interaction domains, which are crucial for both, catalytic and non-catalytic, functions enabling them to act as scaffolds in addition to their catalytic role [209,210].

A variation on this theme is the competition for protein interactions, which can regulate the dynamic turnover of protein complexes. Although an oversimplification, this observation indicates that specificity is mainly achieved by regulating protein interactions that determine the binding of activators and substrates, subcellular localisation and dynamic changes of these conditions. In fact, manipulating protein docking interactions can override the intrinsic catalytic specificity of kinases [211]. Therefore, non-catalytic functions may be paramount to ensure the catalytic specificity of protein kinases. Many of the examples discussed above support this view. Often the cooperation between catalytic and non-catalytic kinase functions is needed to cause the full and correct biological effects. This design also affords maximum versatility by embedding protein kinases into different protein complexes in order to generate different signalling specificities. Pseudokinases may represent the extreme end of this scale. They lack catalytic activity altogether or only exhibit it under highly confined conditions depending on allosteric activation by binding partners [197].

These mechanisms contribute to the specificity of protein kinase action by providing mechanisms of *a priori *substrate discrimination. However, another and complementary way to achieve specificity is to increase the signal to noise ratio of protein kinase signalling. Again, non-catalytic functions seem to be key. An example is provided by the observation that tyrosine kinases often use SH2 domains, which dock to phospho-tyrosines, to recognise substrates previously phosphorylated by themselves or other tyrosine kinases [212]. Many of the signalling complexes discussed above are assembled via non-catalytic kinase functions, but involve multiple positive feedback loops exerted by phosphorylation. The combination of non-catalytic protein complex assembly and catalytic reinforcement of connections or outputs generate a high fidelity filter [147] that can program reliable biological responses.

## Competing interests

The authors declare that they have no competing interests.

## Authors' contributions

JR, NV, DR and WK contributed to the preparation of the manuscript and approval of its final version.
